# Multidrug-Resistant Hypervirulent Group B *Streptococcus* in Neonatal Invasive Infections, France, 2007–2019

**DOI:** 10.3201/eid2611.201669

**Published:** 2020-11

**Authors:** Céline Plainvert, Constantin Hays, Gérald Touak, Caroline Joubrel-Guyot, Nicolas Dmytruk, Amandine Frigo, Claire Poyart, Asmaa Tazi

**Affiliations:** Assistance Publique–Hôpitaux de Paris Centre Université de Paris, Paris, France (C. Plainvert, C. Hays, C. Joubrel-Guyot, N. Dmytruk, A. Frigo, C. Poyart, A. Tazi);; Institut Cochin, Paris (C. Plainvert, G. Touak, C. Poyart, A. Tazi); FHU Prema, Paris (C. Plainvert, C. Poyart, A. Tazi);; Université de Paris, Paris (C. Hays, C. Joubrel-Guyot, C. Poyart, A. Tazi)

**Keywords:** group B *Streptococcus*, *Streptococcus agalactiae*, neonatal infections, hypervirulent CC17 clone, early-onset disease, late-onset disease, bacteria, France, GBS, antimicrobial resistance

## Abstract

We analyzed group B *Streptococcus* (GBS) neonatal invasive infections reported during 2007–2019 in France. The hypervirulent clonal complex (CC) 17 GBS was responsible for 66% (827/1,262) of cases. The role of CC17 GBS increased over time (p for trend = 0.0001), together with the emergence of a multidrug-resistant CC17 GBS sublineage.

Group B *Streptococcus* (GBS; *Streptococcus agalactiae*) is the leading cause of neonatal invasive infections worldwide (*1*). Despite appropriate antimicrobial drug therapy, the global burden of GBS neonatal infections remains substantial, with up to 10% mortality and 30% neurologic sequelae in surviving infants (*2*). Two GBS-associated syndromes are distinguished in neonates: early-onset disease (EOD), which occurs during the first week of life, and late-onset disease (LOD), which occurs after the first week (*1*). In EOD, the neonate is infected by GBS-contaminated maternal secretions during parturition; thus, strategies based on intrapartum antibiotic prophylaxis have drastically diminished its incidence. In contrast, the pathophysiology of LOD remains elusive, and its incidence remains stable (*3**,**4*). Thus, LOD has become the main GBS-associated syndrome in France and other countries in Europe and in North America (*4**,**5*). LOD is largely attributable to a particular GBS clone of serotype III, designated the hypervirulent clonal complex (CC) 17 GBS (*3*,*6*,*7*). Recent epidemiologic data from Canada, China, and Portugal reported the emergence of a multidrug-resistant (MDR) sublineage of CC17 GBS that exhibits acquired nonsusceptibility to 4 antimicrobial categories, namely tetracyclines, aminoglycosides, macrolides, and lincosamides (*8**–**10*). We analyzed neonatal invasive GBS diseases reported to the French National Reference Center for Streptococci during 2007–2019 and investigated the role of the hypervirulent clone over this period.

## The Study

GBS isolates were sent to the National Reference Center by correspondents located throughout the national territory on a voluntary basis. Only invasive infections, such as GBS isolated from a normally sterile site, were considered for this study. A total of 1,262 neonatal invasive infections (EOD, n = 394, 31%; LOD, n = 868, 69%) were reported during 2007–2019. The annual number of cases increased significantly over time as a result of a marked rise in LOD cases since 2013 (Appendix Figure 1). Bacteremia without focus was the main clinical presentation during both EOD and LOD (Table 1). Meningitis represented a frequent complication and was more common in LOD, in which it affected nearly half of infants (p<0.0001; Table 1). The proportion of meningitis during LOD dropped significantly over time, from 69% (95% CI 51%–83%) in 2007 to 33% (95% CI 25%–43%) in 2019 (p for trend = 0.008; Appendix Figure 2). The French recommendations for lumbar puncture indication in neonates did not change during the study period. This observation, together with the increased annual number of cases, suggests a better reporting of bacteremia and a better representativeness of our collection over time.

**Table 1:** Clinical manifestations, serotypes, and CC17 prevalence of group B *Streptococcus* neonatal invasive infections, France, 2007–2019*

Clinical manifestation	EOD, no. (%)	LOD, no. (%)	p value
Bacteremia	298 (75.6)	442 (50.9)	<0.0001#
Ia	69 (23.2)	45 (10.2)	<0.0001**
Ib	13 (4.4)	8 (1.8)	
II	30 (10.1)	6 (1.4)	
III	149 (50.0)	359 (81.2)	
IV	6 (2.0)	6 (1.4)	
V	26 (8.7)	18 (4.1)	
Others†	3 (1.0)	0	
CC17	122 (40.9)	334 (75.6)	<0.0001#
Meningitis‡	95 (24.1)	397 (45.7)	<0.0001#
Ia	15 (15.8)	39 (9.8)	0.33**
Ib	2 (2.1)	12 (3.0)	
II	0	7 (1.8)	
III	74 (77.9)	329 (74.4)	
IV	2(2.1)	2 (0.5)	
V	2 (2.1)	8 (2.0)	
CC17	65 (68.4)	285 (71.8)	0.52#
Others§	1 (0.3)	29 (3.3)	<0.0001#
III	0	24 (82.8)	
Others¶	1	5 (17.2)	
CC17	0	21 (72.4)	
Total	394 (100)	868 (100)	

*CC, clonal complex; EOD, early-onset disease; LOD, late-onset disease. †Including serotypes VI (2 isolates) and VIII (1 isolate). ‡GBS recovered from cerebrospinal fluid (470 cases) or GBS bacteremia associated with a cellular reaction in the cerebrospinal fluid (>20 leukocytes/mm^3^) and consistent clinical findings (4 EOD and 18 LOD cases). §Including bone and joint and skin and soft tissue infections. ¶Including serotypes Ia (3 isolates), Ib (2 isolates), and V (1 isolate). #χ^2^ test for the distribution of the clinical manifestations in EOD and LOD. **χ^2^ test for serotype distribution or CC17 proportion in EOD and LOD during either bacteremia or meningitis.

**Figure 1:**
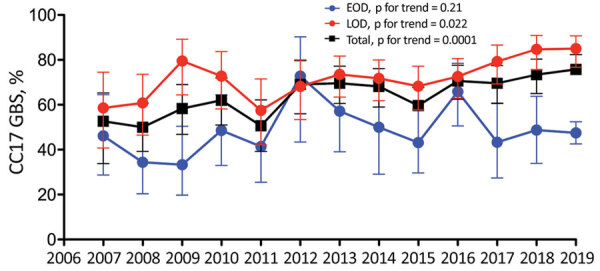
Increasing responsibility of the hypervirulent CC17 clone in GBS neonatal invasive diseases, France, 2007–2019. The annual proportion of infections caused by CC17 GBS during EOD (blue line), LOD (red line), and overall (black line) are represented. Results are expressed as percentage of total GBS isolates per syndrome and per year. Error bars indicate 95% CIs. Evolutionary trends were analyzed using 2-tailed nonparametric Spearman correlation. CC, clonal complex; EOD, early-onset disease; GBS, group B *Streptococcus*; LOD, late-onset disease.

Molecular capsular typing of the 1,262 GBS isolates was performed (*11*) (Table 1). Serotype III was overrepresented, especially in LOD, accounting for 57% (95% CI 52%–62%; n = 223/394) of EOD cases and 82% (95% CI 79%–84%; n = 712/868) of LOD cases. Identification of the hypervirulent CC17 GBS, a highly homogenous CC that includes the sequence type (ST) 17, was performed using a specific PCR (*12*) and showed that it caused 66% (95% CI 63%–68%; n = 827/1,262) of GBS neonatal invasive disease. CC17 GBS prevalence was particularly overwhelming in LOD (74%, 95% CI 71%–77%) compared with EOD (48%, 95% CI 43%–53%; p<0.0001) and, during EOD, in cases of meningitis compared with bacteremia (68%, 95% CI 59%–77% vs. 41%, 95% CI 36%–47%; p<0.0001). Furthermore, CC17 GBS prevalence increased by »50% over the study period, rising from 53% (95% CI 40%–65%) in 2007 to 76% (95% CI 68%–82%) in 2019 (p for trend = 0.0001; Figure 1). This evolution was linked with its prevalence in LOD, which gradually increased from 59% (95% CI 41%–75%) to 85% (95% CI 77%–91%) of the cases during 2007–2019 (p for trend = 0.025).

We determined the susceptibility of the 1,262 GBS isolates to antimicrobial drugs and performed the detection of resistance genes as previously described (*13*). All isolates were susceptible to penicillin, amoxicillin, and vancomycin. Resistance to tetracyclines did not vary through the study period and concerned 91% (95% CI 89%–92%) of the strains, owing to the genetic determinant *tet*(M) in 92% of the cases (data not shown). Only 3 isolates (0.2%, 95% CI 0.1%–0.7%) showed high-level resistance to gentamicin, but high-level resistance to amikacin increased from 0% (95% CI 0%–7%) in 2007 to 18% (95% CI 12%–26%) in 2019 (p for trend <0.0001; Table 2). Similarly, resistance to erythromycin increased from 22% (95% CI 13%–34%) to 30% (95% CI 23%–38%; p for trend = 0.019). Resistance to erythromycin was mostly the result of modifications of the ribosomes that confer cross resistance to lincosamides and are encoded by the genetic determinants *erm*(B) (64%), *erm*(A/TR) (13%), or *erm*(T) (1%), and in 22% of the cases were the result of an efflux mechanism encoded by the genetic determinant *mef*.

**Table 2:** Resistance to erythromycin and high-level resistance to amikacin of GBS neonatal isolates, France, 2007–2019*

Year	Total GBS isolates, resistance, % (95% CI)			CC17 GBS, resistance, % (95% CI)	
	Erythromycin	Amikacin		Erythromycin	Amikacin
2007	21.8 (13.0–34.4)	0.0 (0.0–6.5)		17.2 (7.6–34.6)	0.0 (0.0–11.7)
2008	9.0 (4.4–17.4)	1.3 (0.2–6.9)		5.1 (1.4–16.9)	0.0 (0.0–7.7)
2009	19.4 (12.0–30.0)	1.4 (0.3–7.5)		7.1 (2.5–19.0)	0.0 (0.0–9.0)
2010	21.5 (13.9–31.8)	2.5 (0.7–8.8)		16.3 (8.5–29.0)	0.0 (0.0–8.0)
2011	21.7 (13.6–32.8)	1.5 (0.3–7.8)		8.6 (3.0–22.4)	0.0 (0.0–8.8)
2012	10.9 (5.1–21.8)	3.6 (1.0–12.3)		5.3 (1.5–17.3)	2.3 (0.4–11.8)
2013	17.4 (11.6–25.3)	3.5 (1.4–8.6)		10.0 (5.2–18.5)	0.0 (0.0–4.2)
2014	19.1 (12.8–27.4)	11.8 (7.0–19.2)		10.7 (5.5–19.7)	6.5 (3.0–13.5)
2015	25.6 (18.8–33.7)	14.7 (9.6–21.9)		23.4 (15.3–34.0)	10.6 ((5.7–18.9)
2016	18.4 (12.8–25.7)	11.8 (7.4–18.3)		20.8 (13.9–30.0)	8.4 (4.3–15.7)
2017	25.9 (18.7–34.7)	9.8 (5.6–16.7)		20.5 (13.0–30.8)	9.8 (5.0–18.1)
2018	33.1 (25.4–41.7)	16.9 (11.4–24.5)		29.7 (21.3–39.7)	22.4 (14.8–32.3)
2019	29.7 (22.5–38.1)	18.0 (12.3–25.5)		28.6 (20.6–38.2)	14.1 (9.1–21.1)
p for trend†	0.019	<0.0001		0.0042	<0.0001

*CC, clonal complex; GBS, group B *Streptococcus*. †Evolutionary trends were analyzed using 2-tailed nonparametric Spearman correlation.

**Figure 2:**
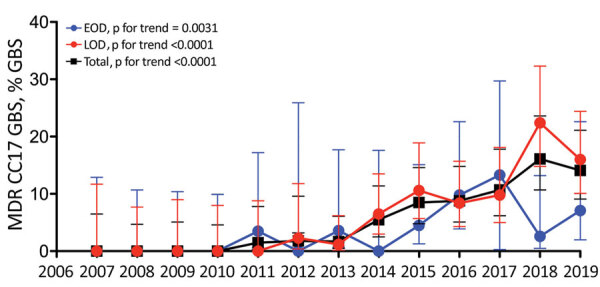
Increasing prevalence of MDR CC17 GBS among neonatal invasive isolates, France, 2007–2019. The annual proportion of infections caused by MDR CC17 GBS, such as those harboring the determinants *tet*(O), *erm*(B), and *aphA-3,* during EOD (blue line), LOD (red line) and overall (black line) are represented. Results are expressed as percentage of total GBS isolates per syndrome and per year. Error bars indicate 95% CIs. Evolutionary trends were analyzed using 2-tailed nonparametric Spearman correlation. CC, clonal complex; EOD, early-onset disease; GBS, group B *Streptococcus*; LOD, late-onset disease; MDR, multidrug-resistant.

Next, we specifically investigated CC17 GBS resistance to erythromycin and amikacin and found an increase over the study period from 17% (95% CI 8%–35%) to 29% (95% CI 21%–38%; p for trend = 0.0042) for erythromycin resistance and from 0% (95% CI 0%–11%) to 14% (95% CI 9%–21%; p for trend <0.0001) for amikacin resistance (Table 2). We postulated that these evolutionary trends were attributable to the emergence of the MDR CC17 GBS sublineage, which exhibits resistance to tetracyclines, macrolides, lincosamides, and amikacin as a result of the replacement of the pilus island 1 genetic locus by mobile genetic elements carrying the resistance determinants *tet*(O), *erm*(B), and *aphA-3* (*8**,**9*). The proportion of CC17 GBS harboring *tet*(O), *erm*(B), and *aphA-3* among neonatal GBS isolates increased from 0% (95% CI 0%–6%) in 2007 to 14% (95% CI 9%–21%) in 2019 (p for trend <0.0001; Figure 2). Whole-genome sequencing of 8 of these MDR CC17 GBS (Appendix Table) confirmed the replacement of the pilus island 1 locus by large integrative and conjugative elements (ICEs) similar to those previously described in China and Canada (*8**,**9*). Interrogation of the ICEberg database (http://db-mml.sjtu.edu.cn/ICEberg/) showed that these ICEs displayed the highest sequence similarity (92%–98%; Appendix Figure 3), with the GBS ICE*Sag*37 described in a CC10 isolate responsible for a neonatal bacteremia in China (*14*).

## Conclusions

We analyzed a total of 1,262 neonatal invasive infections over a 13-year study period in France, which represents »30% of the total national estimated cases (*4*). A selection bias toward the more severe cases cannot be excluded. However, the proportions of EOD and LOD and the associated clinical manifestations described here are very close to the national estimations. Thus, we can assume that our study reflects the national epidemiology without major discrepancies.

We observed a higher reporting of LOD in contrast to EOD over the 13-year study period. This trend mirrors the data from the surveillance network in France, which show a continuous increase in LOD incidence with an overall 65% rise over the past 20 years (*4*). We describe a growing prevalence of the hypervirulent CC17 GBS and of its MDR sublineage in LOD, which might account for the increasing incidence of this syndrome. Whether these trends are the result of a higher tropism of the MDR sublineage for neonatal infections or merely of its selection and clonal expansion as a result of antibiotic selection pressure requires further investigation. Given the worldwide expanding burden of GBS LOD, the adaptability of GBS to its environment through horizontal gene transfer (*15*), and the resulting potential reduction of the therapeutic arsenal against this major neonatal pathogen, our results reinforce the need for a continued surveillance of GBS diseases and for the development of alternative preventive strategies.

Appendix Additional information about multidrug-resistant hypervirulent group B *Streptococcus* in neonatal invasive infections in France.
