# Supplementary material for: Multidrug-Resistant Hypervirulent Group B Streptococcus in Neonatal Invasive Infections, France, 2007–2019
Source: Emerg Infect Dis. 2020 Nov;26(11):2721–4. doi: 10.3201/eid2611.201669 (PMC7588536; doi:10.3201/eid2611.201669)
Supplement: Appendix — Additional information about multidrug-resistant hypervirulent group B Streptococcus in neonatal invasive infections in France. [file 20-1669-Techapp-s1.pdf]

Article DOI: https://doi.org/10.3201/eid2611.201669

Multidrug-Resistant Hypervirulent Group B *Streptococcus* in Neonatal Invasive Infections, France, 2007–2019

Appendix

Appendix Table. Multidrug-resistant CC17 group B *Streptococcus* isolates analyzed by whole genome sequencing in the study of neonatal infections, France*

Bacterial isolate	Year	Source	Onset	NCBI bioproject	NCBI accession no.
CNR_CCH_2011–995	2011	CSF	EOD	PRJNA626549	CP051841
CNR_CCH_2012–845	2012	Blood	LOD	PRJNA626549	CP051842
CNR_CCH_2013–910	2013	Blood	EOD	PRJNA626549	CP051843
CNR_CCH_2013–1366	2013	Blood	LOD	PRJNA626549	CP051844
CNR_CCH_2014–661	2014	Blood	LOD	PRJNA626549	CP051845
CNR_CCH_2018–627	2018	Blood	LOD	PRJNA626549	CP051846
CNR_CCH_2018–670	2018	Blood	LOD	PRJNA626549	CP051847
CNR_CCH_2018–1169	2018	CSF	EOD	PRJNA626549	CP051848

*CSF, cerebrospinal fluid; EOD, early-onset disease; LOD, late-onset disease; NCBI, National Center for Biotechnology Information.

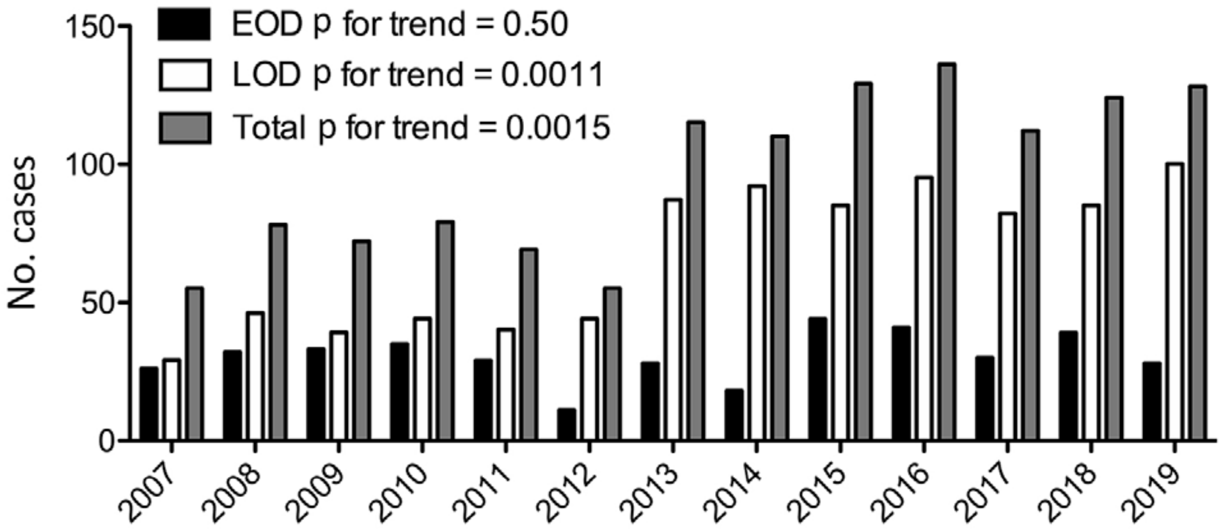

Appendix Figure 1. Group B *Streptococcus* neonatal invasive diseases notified to the National Reference Center from 2007 to 2019. The annual numbers for early-onset disease (EOD; black bars), late-onset disease (LOD; white bars) and total cases (gray bars) are represented. Evolutionary trends were analyzed using 2-tailed nonparametric Spearman correlation.

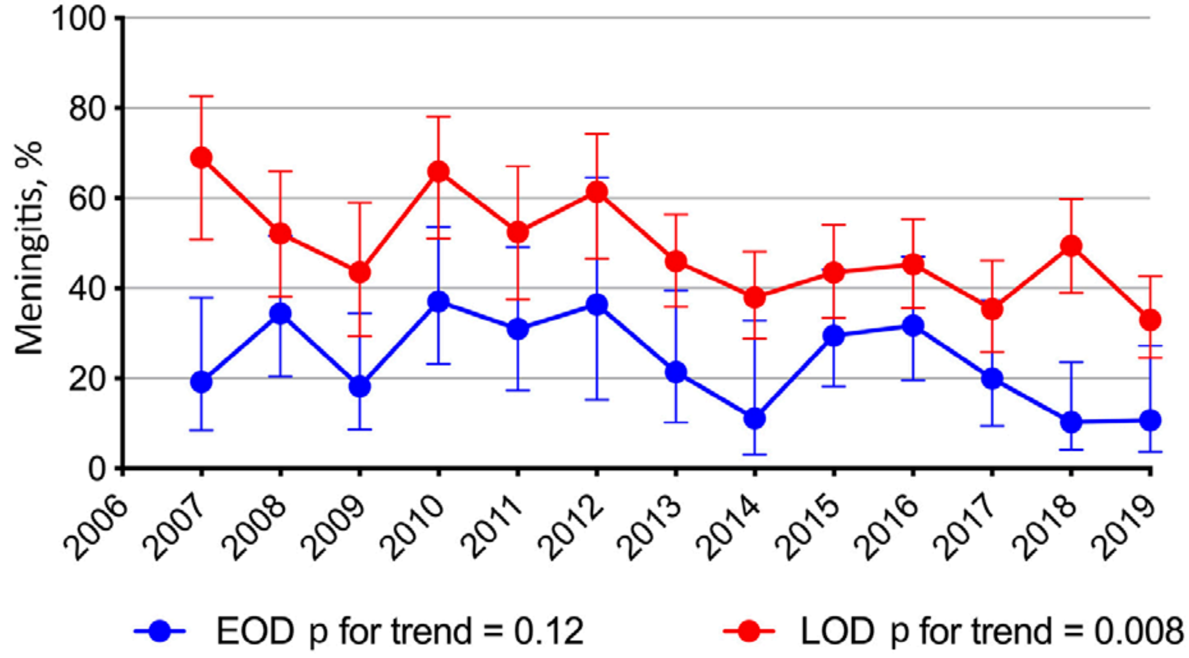

Appendix Figure 2. Group B *Streptococcus* neonatal meningitis cases reported to the National Reference Center, 2007–2019. The annual proportions of meningitis during early-onset disease (EOD; blue line) and late-onset disease (LOD; red line) are represented. Results are expressed as percentage of total invasive cases per syndrome per year. Error bars indicate 95% CI. Evolutionary trends were analyzed using 2-tailed nonparametric Spearman correlation.

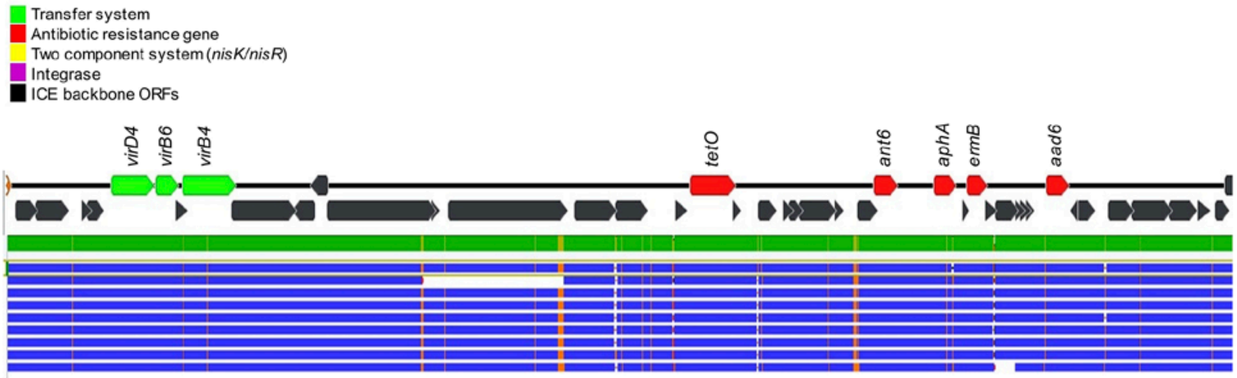

Appendix Figure 3. Sequence comparison of the integrative and conjugative elements (ICEs) identified in the 8 whole-genome–sequenced multidrug-resistant (MDR) CC17 group B *Streptococcus* isolates. Genomes were sequenced using Illumina NextSeq500 instrument (https://www.illumina.com) and assembled with SPADes (Illumina). Sequences were compared by multiple alignments using geneious (https://www.geneious.com). Blue lines depict single ICE sequences. Upper green bar represents the resulting sequence identity (green 100% identity, yellow 80%–99% identity).
